# Carbon, nitrogen, and sulfur elemental and isotopic variations in mouse hair and bone collagen during short-term graded calorie restriction

**DOI:** 10.1016/j.isci.2024.110059

**Published:** 2024-05-22

**Authors:** Eléa Gutierrez, Sharon Mitchell, Catherine Hambly, Kerry L. Sayle, Alex von Kriegsheim, John R. Speakman, Kate Britton

**Affiliations:** 1 Department of Archaeology, University of Aberdeen, Aberdeen, Scotland AB39 2PN, UK; 2 School of Biological Sciences, University of Aberdeen, Aberdeen, Scotland AB24 2TZ, UK; 3 AASPE “Archéozoologie, Archéobotanique: Sociétés, Pratiques, Environnements”, Muséum national d’Histoire naturelle, 75005 Paris, France; 4 Scottish Universities Environmental Research Centre, University of Glasgow, Scotland G75 0QF, UK; 5 Edinburgh Cancer Research UK Centre, MRC Institute of Genetics and Molecular Medicine, University of Edinburgh, Edinburgh Scotland EH4 2XR, UK; 6 Shenzhen Key Laboratory of Metabolic Health, Centre for Energy Metabolism and Reproduction, Shenzhen Institutes of Advanced Technology, Chinese Academy of Sciences, Shenzhen 518055, PRC; 7 Centre of Excellence in Animal Evolution and Genetics, Kunming, PRC; 8 State Key Laboratory of Molecular Developmental Biology, Institute of Genetics and Developmental Biology, Chinese Academy of Sciences, Beijing 100101, PRC

**Keywords:** Archeology, Biochemistry, Isotope chemistry

## Abstract

This study characterized the effect of calorie restriction (CR) on elemental content and stable isotope ratio measurements of bone “collagen” and hair keratin. Adult mice on graded CR (10–40%; 84 days) showed decreased hair *δ*^15^N, *δ*^13^C, and *δ*^34^S values (significantly for *δ*^15^N) with increasing CR, alongside a significant increase in bone “collagen” *δ*^15^N values and a decrease in “collagen” *δ*^13^C values. We propose this was likely due to the intensified mobilization of endogenous proteins, as well as lipids in newly synthesized “collagen”. Elemental analysis of bone “collagen” revealed decreased carbon, nitrogen, and sulfur % content with increasing CR which is attributed to a change in the *in vivo* bone “collagen” structure with extent of CR. This complexity challenges the use of elemental indicators in the assessment of collagen quality in archaeological studies where nutritional stress may be a factor.

## Highlights

• Tissues sampled from mice following graded calorie restriction (CR) study
• Bone “collagen” *δ*^15^N increased with extent of CR, but *δ*^13^C decreased
• C, N, and S content (%) measurements of bone “collagen” decreased with CR extent
• Keratin data may reflect slower hair growth/increased hair retention with CR extent

## Graphical abstract

**Figure:**
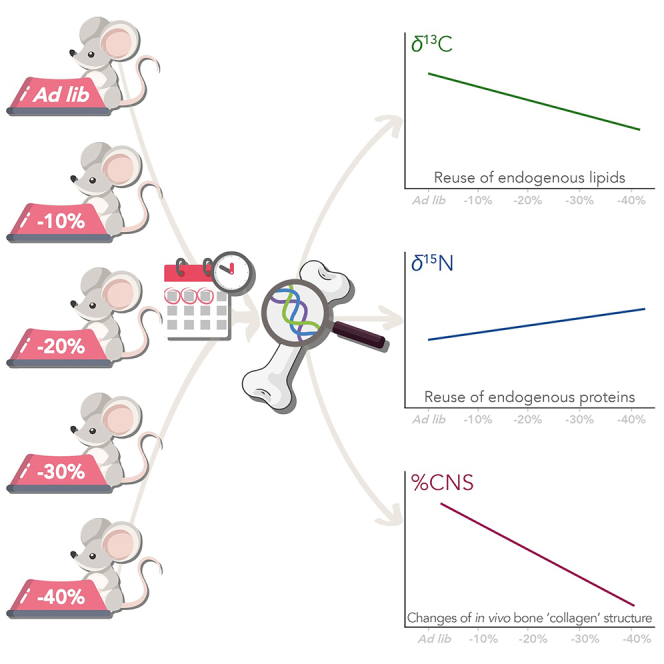

## Introduction

The stable isotope analysis of different tissues is commonly employed in both archaeology and ecology to address diverse questions relating to individual and group diet, movements, and other aspects of life histories.^1^^,^^2^^,^^3^^,^^4^^,^^5^ Determination of trophic position continues to be one of the main applications in these disciplines. In ecology, knowing trophic position allows better understanding of species interactions.^6^ In archaeology, identification of the main sources of protein consumed during life permits us to better understand the dietary habits of past populations,^7^^,^^8^^,^^9^ dietary changes in major time transitions,^10^^,^^11^ changes in individual diet during life,^12^^,^^13^^,^^14^ the life history of a single individual,^15^ infant feeding practices,^16^^,^^17^^,^^18^ and dietary variations within key aspects of socio-cultural identities.^19^^,^^20^^,^^21^^,^^22^ While carbon and nitrogen isotope analyses are most commonly employed, the use of other isotopes for palaeodietary studies is increasing, such as oxygen,^23^^,^^24^^,^^25^ strontium,^26^ zinc,^27^^,^^28^ and sulfur.^29^^,^^30^^,^^31^ Carbon and nitrogen are among the best understood in terms of their underlying principles given the frequency of their use and long history of their application. For example, isotopic fractionation effects are well defined for the most frequently used tissues in ecology (e.g., blood, urine, liver, and muscle)^32^^,^^33^ and in archaeology (e.g., bone and hair).^34^^,^^35^ More research into other elements, such as sulfur, is however required and, in the case of sulfur, both ecologists^36^ and archaeologists^30^ have been working on its characterization. There is also a growing literature exploring the influence different factors may have on isotopic fractionation, such as growth,^37^^,^^38^ water stress,^34^^,^^39^ gestation,^40^^,^^41^ during disease states,^42^^,^^43^^,^^44^ and nutritional stress.^45^^,^^46^^,^^47^^,^^48^^,^^49^ The latter is inadequately characterized, despite the numerous feeding experiments to determine this isotopic fractionation for carbon and nitrogen.^39^^,^^45^^,^^47^^,^^48^^,^^50^^,^^51^

In nutritional biology and ecology, calorie restriction (CR) is studied to facilitate better understanding of its effect on lifespan and animal physiology, for example during natural fasts^52^ or in a pathological context.^48^^,^^53^ In archaeology, the potential to identify and characterize past nutritional stress on individual and population levels using stable isotope analyses has great potential to illuminate past living conditions, individual health, socio-economic status, and past eco-political conditions (e.g., famines). However, isotopic studies carried out so far have mainly been undertaken by ecologists and have focused on soft tissues that are rarely preserved in archaeological contexts. While a number of studies have included hair keratin,^53^^,^^54^^,^^55^ which is sometimes involved in archaeological studies, few studies have focused on bone collagen,^47^ (herein referred to as “collagen”). While the methodology used to extract bone collagen is well-established (the Longin methodology^56^) and commonly used across archaeology and palaeontology, it is well-known that—while type-1 collagen predominates—it is usual that other proteins are also extracted during the process such as small proportions of other types of collagen or proteoglycans.^57^ However, the term collagen (sometimes written as “collagen”) is usually used across both these fields (and others) to refer protein extracted from bone for bulk isotopic analysis (as opposed to compound-specific approaches, etc.).^58^

Combined analyses of these two tissues (bone “collagen”, hair keratin) in a controlled CR experiment has not previously been published and there is a lack of understanding of how carbon and nitrogen isotope values vary between these two tissues during dietary stress. This combination would enhance the possibility of comparing modern and archaeological samples, as these tissues are most commonly preserved in the archaeological record.^59^ Also, despite the growing number of experimental studies using CR, none incorporating isotope analyses have been conducted using graded levels of restriction.

Furthermore, elemental compositions are rarely reported in previous studies using stable isotopes^60^ or utilized in interpretations.^61^^,^^62^ In archaeology, the contents of carbon (%C), nitrogen (%N), and sulfur (%S), as well as the carbon-to-nitrogen ratio (C:N) are important in defining the quality of the tissues analyzed and to identify samples that may have been contaminated in the burial environment or undergone diagenetic alteration. While it has been previously observed that the elemental composition of “collagen” can vary within and between species,^29^^,^^63^^,^^64^ the values have been determined based on amino acid composition of “collagen” from animals in good condition (i.e., no physiological stress). However, given that starvation can cause skeletal changes (e.g., bone loss),^65^^,^^66^ and that starvation has been demonstrated to induce changes in the hair composition,^67^^,^^68^ the influence of CR on the elemental composition of bone and hair (and by extension on the definition of quality indicators) requires further consideration. To date there have been no studies of animals on a single diet to check whether %C, %N, and %S change with the physiological state of the individual.

Based on data obtained from previous studies, we can expect that *δ*^15^N values will increase during nutritional stress due to the recycling of endogenous proteins that undergo a second isotope fractionation process.^45^^,^^47^^,^^69^ This increase has been observed in various tissues and species (e.g., bone collagen in rodents, muscle, or liver in avians). To the contrary, *δ*^13^C values should decrease due to a more intensive use of lipids during dietary stress as it has been observed in human hair for instance.^53^^,^^54^ However, these variations are still debated,^39^^,^^48^^,^^70^ and these variable results have been explained by differential turnover between tissues^39^ or using levels of restriction that were insufficient to see effects.^71^ No data are available for sulfur stable isotope values. The aim of this study was therefore to characterize the isotopic and elemental variations of carbon, nitrogen, and sulfur in a context of graded CR (up to 40%) on bone “collagen” and hair keratin.

## Results

### Stable isotopic values of the diet

**Figure 1:**
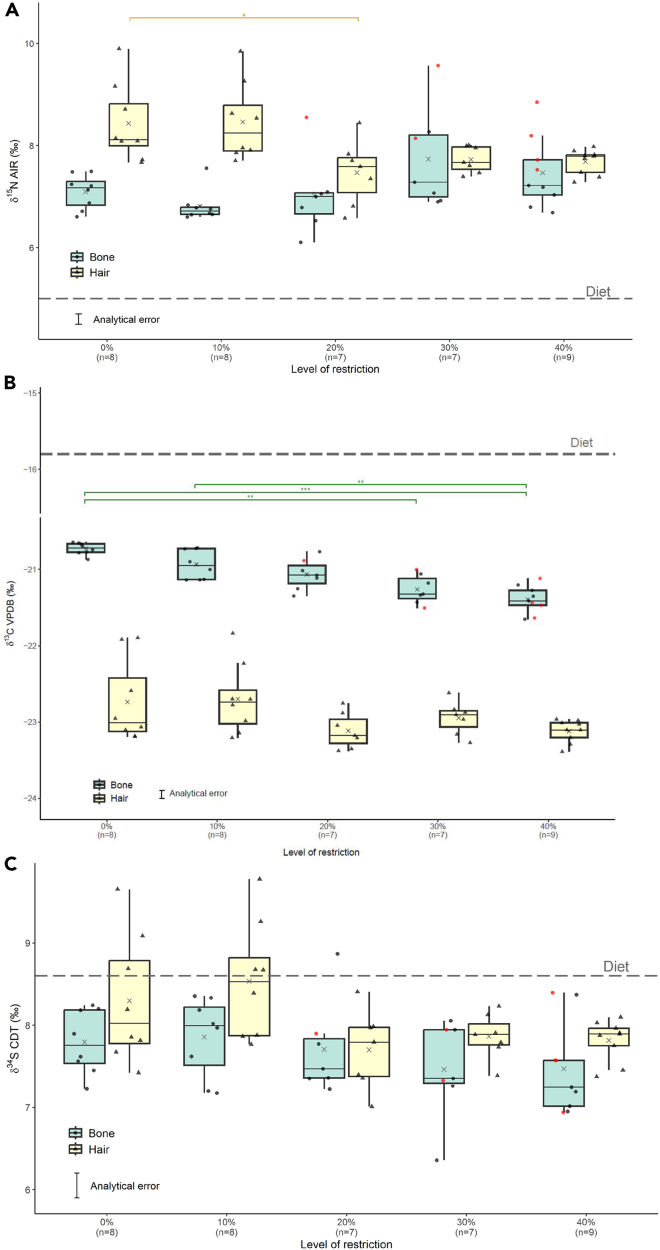
Distribution of isotopic values from mice undergoing three months graded calorie restriction (CR) for bone (in green) and hair (in yellow) Distribution of *δ*^15^N values (A), *δ*^13^C values (B) and *δ*^34^S values (C) in mice undergoing a graded calorie restriction (CR) of the same diet by a 0%, 10%, 20% and 40% reduction of *ad libitum* intake for a period of three months. The gray crosses show the mean values for each group, and the horizontal lines through the boxes represent the median. The long-dashed line represents the value for the diet during the experiment. The red dots represent mice (included in the analysis) that did not meet the accepted quality criteria for C:N_coll_ according to Ambrose^72^ and will be discussed in a subsequent section. ∗, ∗∗, and ∗∗∗ indicate a significant difference between groups with a *p* value < 0.025, <0.01, and <0.001 respectively.

**Figure 2:**
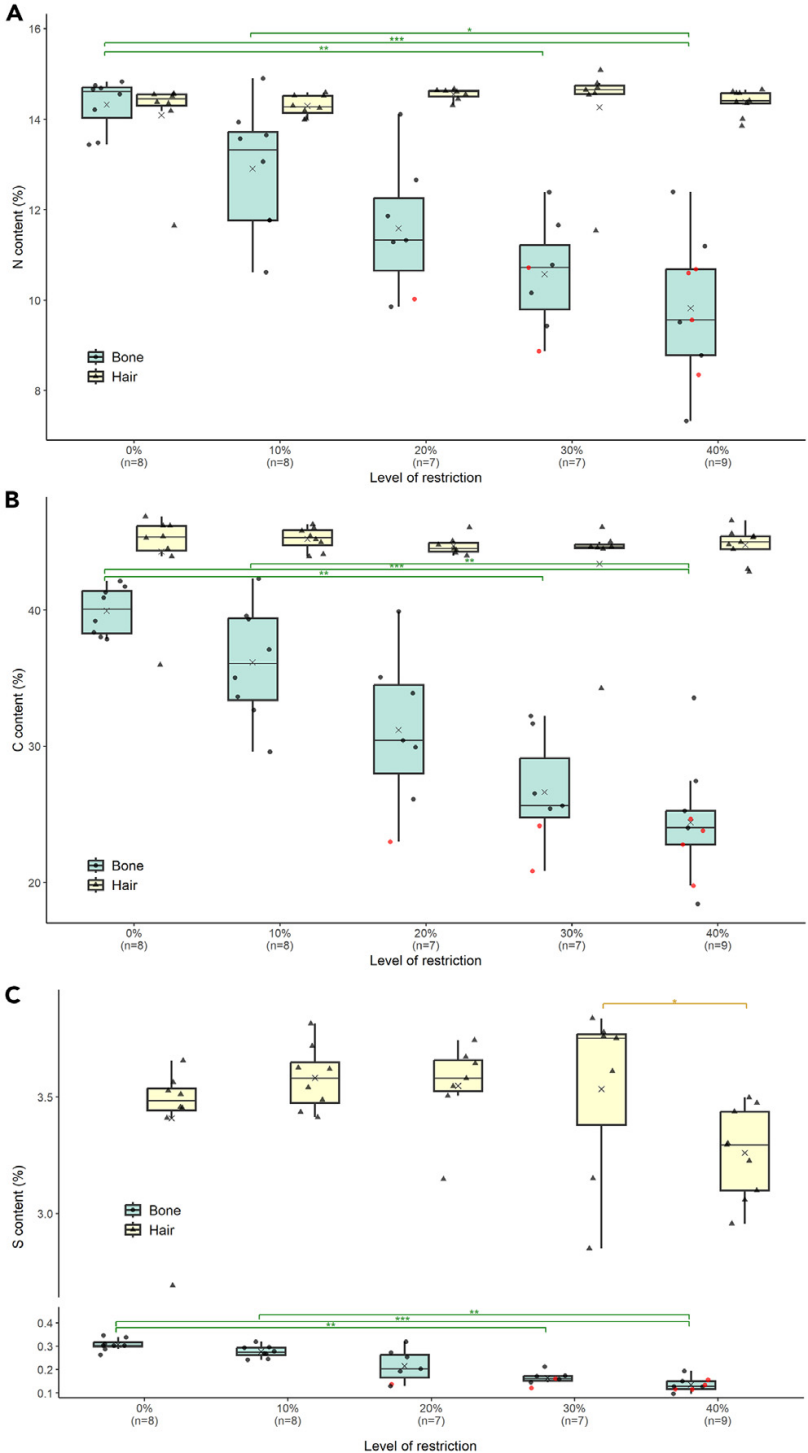
Distribution of elemental composition from mice undergoing three months graded calorie restriction (CR) for bone (in green) and hair (in yellow) Distribution of %N (A), %C (B) and %S (C) in mice undergoing a graded calorie restriction (CR) of the same diet by a 0%, 10%, 20% and 40% reduction of *ad libitum* intake for a period of three months. The gray crosses indicate the mean values for each group, and the horizontal lines through the boxes represent the median. The red dots represent mice (included in the analysis) that did not meet the accepted quality criteria for C:N_coll_ according to Ambrose.^72^ ∗, ∗∗, and ∗∗∗ indicate a significant difference between groups with a *p* value < 0.025, <0.01, and <0.001 respectively.

**Figure 3:**
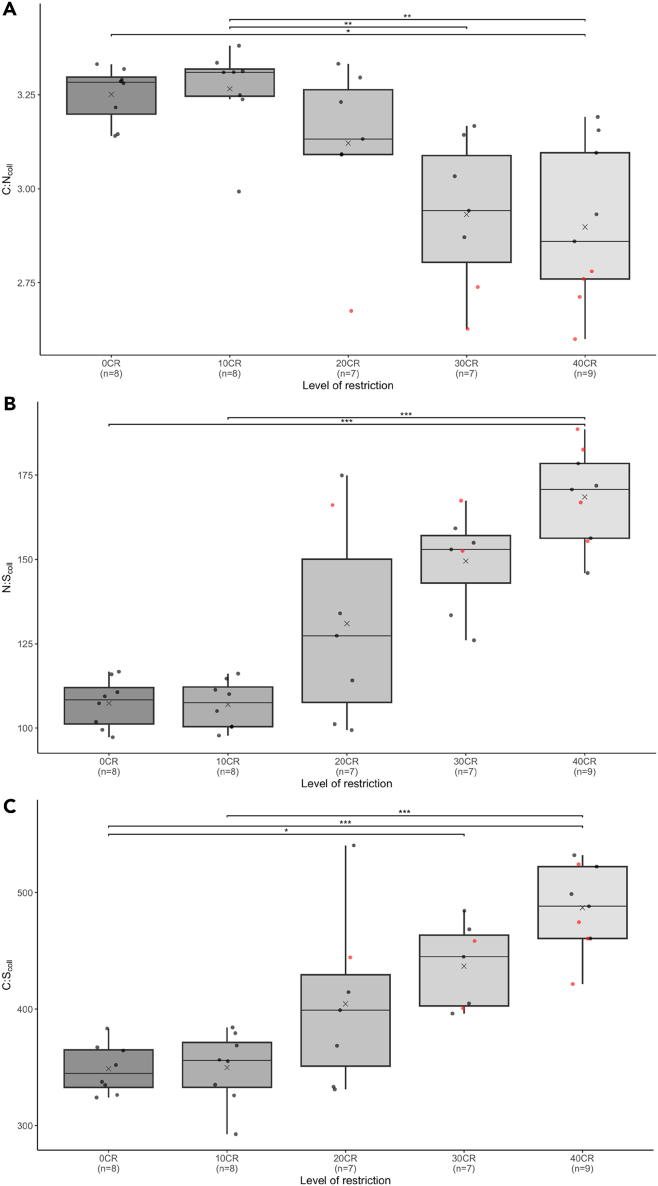
Distribution of elemental ratio in bone “collagen” from mice undergoing three months graded calorie restriction (CR) Distribution of carbon-to-nitrogen ratio (C:N) (A), nitrogen-to-sulfur ratio (N:S) (B) and carbon-to-sulfur ratio (C:S) (C) in mice undergoing a graded calorie restriction (CR) of the same diet by a 0%, 10%, 20% and 40% reduction of *ad libitum* intake for a period of three months. The gray crosses indicate the mean values for each group, and the horizontal lines through the boxes represent the median. The red dots represent mice (included in the analysis) that did not meet the accepted quality criteria for C:N_coll_ according to Ambrose.^72^ ∗, ∗∗, and ∗∗∗ indicate a significant difference between groups with a *p* value < 0.025, <0.01, and <0.001 respectively.

Isotopic and elemental data for all individuals are provided in Tables 1 and 2, and Supplementary Information 1, and in Figures 1, 2, and 3. The rodent diet (D12450B, Research diets, NJ, USA) isotopic values were 5.0‰ for *δ*^15^N_diet_, −15.8 ‰ for *δ*^13^C_diet_, and 8.6 ‰ for *δ*^34^S_diet_. Nitrogen and sulfur mainly come from proteins, while carbon mostly comes from carbohydrates and fats. However, the diet was analyzed whole, and the isotope ratios of the protein, carbohydrate, and fat portions of the diet were not analyzed separately, Δ^13^C_collagen-diet_ values were not further considered here. To avoid the effect of CR, only the 0% group was discussed regarding offsets between tissue and diet and tissue to tissue.

**Table 1:** Stable isotopic data of bone “collagen” (coll) and hair keratin (ker) from mice undergoing three months of graded calorie restriction (CR)

	CR group (n)	0% (8)	10% (8)	20% (7)	30% (7)	40% (9)
*δ*^15^N_coll_	Mean	7.1	6.8	7.0	7.7	7.5
	Median	7.2	6.7	7.0	7.3	7.2
	IQR	0.46	0.14	0.42	1.21	0.68
	Range	6.6–7.5	6.6–7.6	6.1–8.6	6.9–9.6	6.7–8.9
*δ*^15^N_ker_	Mean	8.4	8.5	7.5	7.7	7.7
	Median	8.1	8.2	7.6	7.7	7.8
	IQR	0.83	0.89	0.68	0.44	0.34
	Range	7.7–9.9	7.7–9.9	6.6–8.4	7.4–8.0	7.3–8.0
Δ^15^N_coll-diet_	Mean	2.1	1.8	2.0	2.7	2.5
	Median	2.2	1.7	2.0	2.3	2.2
	IQR	0.46	0.14	0.42	1.21	0.68
	Range	1.6–2.5	1.6–2.6	1.1–3.6	1.9–4.6	1.7–3.9
Δ^15^N_ker-diet_	Mean	3.4	3.5	2.5	2.7	2.7
	Median	3.1	3.2	2.6	2.7	2.8
	IQR	0.83	0.89	0.68	0.44	0.34
	Range	2.7–4.9	2.7–4.9	1.6–3.4	2.4–3.0	2.3–3.0
Δ^15^N_coll-ker_	Mean	−1.3	−1.7	−0.5	0.01	−0.2
	Median	−1.0	−1.6	−0.5	−0.1	−0.3
	IQR	0.93	0.82	0.56	1.31	0.58
	Range	−2.7/−0.6	−3.0/−0.4	−1.0/0.1	−1.1/1.9	−1.1/0.9
*δ*^13^C_coll_	Mean	−20.7	−20.9	−21.1	−21.3	−21.4
	Median	−20.7	−21.0	−21.1	−21.3	−21.4
	IQR	0.11	0.4	0.23	0.26	0.2
	Range	−20.9/−20.6	−21.1/−20.7	−21.4/−20.8	−21.5/−21.0	−21.6/−21.1
*δ*^13^C_ker_	Mean	−22.7	−22.7	−23.1	−23.0	−23.1
	Median	−23.0	−22.7	−23.2	−22.9	−23.1
	IQR	0.7	0.44	0.32	0.21	0.19
	Range	−23.2/−21.9	−23.2/−21.8	−23.4/−22.8	−23.3/−22.6	−23.4/−23.0
Δ^13^C_coll-ker_	Mean	2.0	1.8	2.1	1.7	1.7
	Median	2.3	1.9	2.0	1.7	1.8
	IQR	0.82	0.65	0.28	0.3	0.31
	Range	1.2–2.5	1.1–2.3	1.8–2.6	1.4–2.0	1.3–2.2
*δ*^34^S_coll_	Mean	7.8	7.9	7.7	7.5	7.5
	Median	7.8	8.0	7.5	7.4	7.3
	IQR	0.65	0.71	0.48	0.65	0.56
	Range	7.2–8.2	7.2–8.4	7.2–8.9	6.4–8.1	6.9–8.4
*δ*^34^S_ker_	Mean	8.3	8.6	7.7	7.9	7.8
	Median	8.0	8.5	7.8	7.9	7.9
	IQR	1.01	0.95	0.6	0.25	0.21
	Range	7.4–9.7	7.8–9.8	7.0–8.4	7.4–8.2	7.4–8.1
Δ^34^S_coll-diet_	Mean	−0.8	−0.7	−0.9	−1.1	−1.1
	Median	−0.8	−0.6	−1.1	−1.3	−1.4
	IQR	0.65	0.71	0.48	0.65	0.56
	Range	−1.4/−0.4	−1.4/−0.3	−1.4/0.3	−2.2/−0.5	−1.7/−0.2
Δ^34^S_ker-diet_	Mean	−0.3	−0.1	−0.9	−0.7	−0.8
	Median	−0.6	−0.1	−0.8	−0.7	−0.7
	IQR	1.01	0.95	0.6	0.25	0.21
	Range	−1.2/1.1	−0.8/1.2	−1.6/−0.2	−1.2/−0.4	−1.2/−0.5
Δ^34^S_coll-ker_	Mean	−0.5	−0.7	0.01	−0.4	−0.3
	Median	−0.3	−0.7	−0.4	−0.6	−0.3
	IQR	0.89	1.33	0.82	0.67	1.21
	Range	−2.1/−0.8	−1.6/0.6	−0.6/1.5	−1.0/0.3	−1.1/0.6

Mice were restricted by 0–40% *ad libitum* intake. Group means, medians, and ranges are reported in ‰. IQR refers to interquartile ranges.

**Table 2:** Elemental data of hair keratin (ker) and bone “collagen” (coll) from mice undergoing three months of graded calorie restriction (CR)

	CR group (n)	0% (8)	10% (8)	20% (7)	30% (7)	40% (9)
%N_ker_	Mean	14.1	14.3	14.6	14.3	14.4
	Median	14.4	14.3	14.6	14.6	14.4
	IQR	0.24	0.37	0.13	0.19	0.22
	Range	11.6–14.6	14.0–14.6	14.3–14.7	11.5–15.1	13.9–14.7
%C_ker_	Mean	44.3	45.2	44.7	43.4	44.8
	Median	45.4	45.3	44.5	44.6	45.0
	IQR	1.84	1.11	0.64	0.29	0.93
	Range	36.0–46.9	43.9–46.3	44.0–46.1	34.3–46.1	42.8–46.6
%S_ker_	Mean	3.4	3.6	3.6	3.5	3.3
	Median	3.5	3.6	3.6	3.8	3.3
	IQR	0.09	0.17	0.13	0.39	0.34
	Range	2.7–3.7	3.4–3.8	3.2–3.7	2.9–3.8	3.0–3.5
%N_coll_	Mean	14.3	12.9	11.6	10.6	9.8
	Median	14.6	13.3	11.3	10.7	9.6
	IQR	0.67	1.96	1.60	1.42	1.90
	Range	13.4–14.8	10.6–14.9	9.9–14.1	8.9–12.4	7.3–12.4
%C_coll_	Mean	40.0	36.2	31.2	26.6	24.4
	Median	40.1	36.1	30.4	25.6	24.0
	IQR	3.15	6.0	6.45	4.32	2.46
	Range	37.9–42.1	29.6–42.3	23.0–39.9	20.8–32.2	18.4–33.5
%S_coll_	Mean	0.31	0.28	0.22	0.16	0.14
	Median	0.30	0.27	0.20	0.16	0.13
	IQR	0.02	0.03	0.1	0.02	0.03
	Range	0.26–0.35	0.24–0.32	0.13–0.32	0.12–0.21	0.10–0.19
C:Ncoll	Mean	3.3	3.3	3.1	2.9	2.9
	Median	3.3	3.3	3.1	2.9	2.9
	IQR	0.1	0.07	0.17	0.28	0.34
	Range	3.1–3.3	3.0–3.4	2.7–3.3	2.6–3.2	2.6–3.2

Mice were restricted by 0–40% *ad libitum* intake. Group means, medians and ranges are reported in ‰. IQR refers to interquartile ranges.

### Nitrogen isotopic values for bone and hair

Nitrogen isotope ratios of extracted bone protein (“collagen”) were statistically higher in the most restricted groups (30% and 40%) compared to the control group (0%, Kruskal-Wallis, *H*_(4)_ = 10.596 *p* < 0.05) (Table 1). However, because of the lower threshold of the null hypothesis rejection, post-hoc Dunn test showed that none of the groups were different from the others.

The *δ*^15^N_ker_ values decreased from the 0% and 10% groups to the most restricted groups (20%, 30%, 40%), which was statistically significant (Kruskal-Wallis, *H*_(4)_ = 14.596 *p* < 0.01) (Table 1, and Figure 1A). However, only the 0% and 20% groups were statistically different (Dunn test, *p* < 0.025). Bone extract and hair keratin were ^15^N-enriched compared to the diet, while keratin was ^15^N-enriched relative to bone protein. Correlation between keratin-collagen intra-individual pairs for the control group was not statistically significative (Spearman, ρ = 0.31, *p* = 0.46) (Figure S1). Correlation between mass gain (between start and end of the CR experiment) and the nitrogen offset between bone protein and diet of the control group was also not statistically significant (Spearman, ρ = 0.0, *p* = 1) (Figure S2).

### Carbon isotopic values for bone and hair

The *δ*^13^C_coll_ values decreased in a graded manner with increasing restriction level (Kruskal-Wallis, *H*_(4)_ = 25.911 *p* < 0.0001) (Figure 1B and Table 1). The 0% group was significantly different from the 30% and the 40% group (Dunn, *p* < 0.01). The 10% group was also significantly different from the 40% group (*p* < 0.01).

Unlike bone “collagen”, hair keratin *δ*^13^C_ker_ values did not vary significantly between feeding levels (Kruskal-Wallis, *p* > 0.05). However, *δ*^13^C_ker_ mean values decreased slightly (and beyond the analytical error of 0.1‰) (Figure 1B) with a difference of 0.4‰ between the 0% and 40% groups (Table 1), while the *δ*^13^C_ker_ median values did not vary between groups. Bulk bone protein was ^13^C-enriched compared to keratin.

### Sulfur isotopic values for bone and hair

In contrast to nitrogen and carbon isotope ratios, the *δ*^34^S_coll_ values did not vary significantly with CR (Kruskal-Wallis, *p* > 0.05). The mean difference between the 0% group and the 40% group is within the analytical error, while the median differed of 0.5 ‰ (Figures 1C and Table 1). There was a trend of decreasing *δ*^34^S_ker_ mean values with CR which did not reach significance (Kruskal-Wallis, *p* > 0.05), while the median did not vary (Table 1 and Figure 1C). The diet was ^34^S-enriched in relation to bone extract and hair keratin, while hair keratin was ^34^S-enriched compared to bone “collagen” (Table 1).

### Hair and bone elemental data for C, N, and S

The nitrogen and carbon contents for hair keratin did not vary significantly with CR level (Kruskal-Wallis, *p* > 0.05). While CR did not impact %N_ker_ and %C_ker_, %S_ker_ varied significantly with CR (Kruskal-Wallis, *H*_(4)_ = 11.658 *p* < 0.05). Post-hoc Dunn tests showed that the 40% group differed from the 10% group and the 30% group (*p* < 0.025) affecting the variation in C:S_ker_ and N:S_ker_ (Kruskal-Wallis, *p <* 0.01) (Figure S3).

In contrast to hair, there was a graded reduction in elemental composition of bone extracts for all three elements as the level of restriction increased (Figure 2). %N_coll_, %C_coll_, and %S_coll_ almost halved from the 0% group to the 40% group (Table 2). All three elements responded significantly to CR (Kruskal-Wallis, *p* < 0.0001). Post-hoc Dunn tests showed that for all three elements, the group 0% differed from the groups 30% and 40% while the group 10% differed from the group 40% (*p* < 0.025).

Not only did %C, %N, and %S differ with extent of CR, but C:N_coll,_ N:S_coll_, and C:S_coll_ also differed according to the degree of CR (Kruskal-Wallis, *p* < 0.001; normality of the variable checked using a Shapiro-Wilk test, *p* < 0.05) with the 0% group being significantly different from the 40% group, and the 10% group being significantly different from the 30% and 40% groups (Dunn, *p* < 0.025) for C:N_coll_. The 40% group is significantly different from the 0% and 10% (Dunn, *p* < 0.001) for N:S_coll_, while the 0% group is different from the 30% and 40% groups, and the 10% group is different from the 40% group (Dunn, *p* < 0.025) for C:S_coll_ (Figure 3).

According to published quality indicators (i.e., the compositional measurements used widely in archaeology to assess the preservation of bone collagen, including %C, %N, C:N, etc.),^72^ seven individuals (highlighted in red in Figures 1, 2, and 3) were outside the range for C:N_coll_ including four in the 40% group (around 44 % of the group), two in the 30% group (around 29 % of the group) and one in the 20% group (around 14 % of the group) with a C:N_coll_ between 2.6 and 2.8. Whereas in an archaeological dataset it would be recommended to remove these individuals because of potential contamination or diagenetic alteration, in this modern dataset the decision has been taken to keep them and discuss their deviance. As shown in Figure 1, individuals with C:N below 2.9 had *δ*^15^N_coll_, *δ*^13^C_coll_, and *δ*^34^S_coll_ values distributed around the mean for their group.

To assess the relationship between the isotopic values and bone elemental content, Spearman’s rank correlations were applied. The *δ*^34^S_coll_ and %S_coll_ values were not correlated (ρ = 0.19, *p* = 0.2), but the *δ*^15^N_coll_ and %N_coll_, and the *δ*^13^C_coll_ and %C_coll_ values were (*p* < 0.05) with a coefficient (ρ) of −0.3 and 0.7, respectively. For a more precise look at these correlations, Spearman’s rank correlations between each group were performed. Only the *δ*^13^C_coll_ and %C_coll_ values of the 10% group were correlated (ρ = 0.80, *p* = 0.02). Furthermore, the correlation was not consistent between the groups (Figure S4).

### Proteomic analysis

Apart from one sample (ST-50), the 15 samples analyzed (relative proportion of collagen type-I can be found in Table S3) were determined to be predominantly collagen type-I (including α-1 and α-2 chains). Removing this outlier (ST-50), the average of the samples is 76 % collagen with a range from 61 % to 92 %. For the outlier, myosin was determined to be the main protein component. Six of the seven individuals (highlighted in red in Figures 1, 2, and 3) were included in the proteomic analysis. Their relative proportions of collagen type-I were not different from the rest of the subsample (between 62 % and 89 % of collagen type-I). For the whole subsample, both elemental content and stable isotope values were within the range of the group to which it belonged. No correlation was detected between the relative proportion of collagen and elemental data, nor isotopic values (Spearman, *p* > 0.05). There was also no statistical difference (ANOVA, *p* > 0.05) between the different groups for the relative proportion of collagen.

## Discussion

### Diet-tissue and inter-tissue trends

It is worth noting that prior to the mice being purchased (up to 12 weeks of age), they were fed a different diet (the standard Charles River Laboratories mouse diet) during their growth in the facility where they were born. This Charles River diet was not obtained at the time of the feeding experiment and could not therefore be isotopically characterized. However, a sample of this soya-based animal house diet was obtained in 2022 (*δ*^15^N_CharlesRiverdiet_ = 2.5 ‰). It should be noted that—although dietary composition had not changed—the source/origin of ingredients might have changed over the 13 years between those animals being raised and this study.

#### Inter-tissue offsets

The trophic offset between keratin and collagen is supposedly negligible in rodents.^39^ Given the low *δ*^15^N and *δ*^34^S values of the measured pre-experimental food sample, it may explain the negative Δ^15^N_coll-ker_ and Δ^34^S_coll-ker_ values in the non-restricted (0%) group. The absence of correlation between keratin-collagen intra-individual pairs in the control group for *δ*^15^N values shows that equilibrium is not reached for one of the tissues yet. In contrast, the Δ^13^C_coll-ker_ value is closer to what is expected^73^ perhaps due to the quicker turnover for carbon compared to nitrogen.^74^ It is also important to highlight that chemical structure of hair keratin and bone collagen differ (i.e., methionine is the only sulfur-containing amino acid for bone collagen, while hair keratin has a combination of methionine and cysteine). Thus, sulfur in hair may originate either from dietary origin or endogenous cysteine-containing proteins (i.e., free cysteine). These two origins may exhibit different values due to the diet-switch at 12 weeks of age, leading to a variability in the offsets of the two tissues. Furthermore, while hair does grow, once grown hair is metabolically inert,^75^^,^^76^ which means that the isotope values in hair do not change after tissue formation until that tissue is lost through shedding. Bone, on the other hand, is remodeled throughout life, and isotope values often represent an average of the years before the individual’s death or even the entire lifetime diet,^77^^,^^78^ which leads to variability in tissue-to-tissue offsets.

#### Diet-tissue equilibrium

Besides the differences between the tissues, there are also anticipated offsets between diet and the two tissues. Offsets between bone “collagen” and diet (Δ^15^N_coll-diet_) are normally defined between +3 ‰ and +5 ‰^34^^,^^79^^,^^80^ whereas a Δ^15^N_coll-diet_ of 2.2 ‰ was measured in the unrestricted group. When the bones were collected, the mice had been fed the experimental diet (D12450B) for five months (i.e., from three months of age upon arrival at the University of Aberdeen). The bone “collagen” isotope values were probably also affected by the Charles River diet and the results from the bone extract analysis perhaps evidence their transition to the newer (i.e., experimental) diet. Some studies showed that cranium had a lower bone turnover rate than long bone,^81^^,^^82^^,^^83^ although others suggest the contrary.^84^^,^^85^ Given that cancellous bone in femur in mice has a turnover rate of ca. 0.7 % per day,^86^^,^^87^ it is expected that full renewal of the cranium would take more than ca. 4.7 months. A Δ^15^N_coll-diet_ value below the expected offset between bone “collagen” and diet is thus expected, suggesting a mixture of old and new collagen. Furthermore, the absence of correlation between mass gain and the offset between bone protein and diet for nitrogen strengthen our hypothesis of the near-equilibrium between diet and tissue. Considering the offset between keratin and bone “collagen” for carbon (2.3 ‰ for the control group), this seems to confirm that bone “collagen” was close to equilibrium with the experimental diet.^59^ In contrast, the observed offsets between the diet and keratin for *δ*^15^N values were more typical (3.1 ‰ for the control group) and consistent with previously published results (e.g., 3.3 ‰ in rodents^39^).^79^^,^^88^^,^^89^^,^^90^ Furthermore, the hair follicle cycle takes approximately three weeks in mice.^91^ This suggests that after five months on the experimental diet, keratin *δ*^15^N was closer to (or had reached) equilibrium with the experimental diet.^59^ Regarding sulfur isotopes, the offset between diet and tissues is also in the expected range (i.e., ±0.5 ‰ for“collagen”,^92^ and −1 ‰ for hair keratin.^30^

### Variation in isotope ratios of keratin with extent of CR

The similarity between the 0% and 10% groups for *δ*^15^N_ker_, *δ*^13^C_ker_, and *δ*^34^S_ker_ values was consistent with the fact that mice in the 10% group were able to quickly return to energy balance. A loss of both fat mass and fat-free mass was detected for groups with CR of 20% or greater, while no loss of either was detected in the 10% group through DXA (dual-energy X-ray absorptiometry) analyses,^93^ corroborating this hypothesis.

Although both bone collagen and keratin are synthesized from amino acids within the bodily pool because hair keratin is (anticipated to be) constantly growing but not undergoing turnover, it can be expected that stable isotopes in newly synthesized keratin would reflect variability related to diet or CR more quickly than bone collagen (which constantly remodels slowly). Our analysis revealed distinct nitrogen offsets between keratin and diet in the 0% and 10% CR groups (e.g., Δ^15^N_ker-diet_ of 3.1 ‰ for the control group), while the more restricted groups (20%, 30%, and 40%) exhibited slightly lower offsets (e.g., Δ^15^N_ker-diet_ of 2.8 ‰ for the 40% group), suggesting incomplete equilibration of hair with the new diet for the most calorie restricted mice. Depending on the specific developmental stage of the hair (e.g., anagen, telogen), the hair follicle attains a level of maturation at which CR does not influence its growth, whereas newly synthesized hair could exhibit a comparatively decelerated growth rate.^94^^,^^95^ Given the notably low estimated *δ*^15^N values (2.5 ‰) of the Charles River diet, it is plausible that hair in the most restricted group required more time to achieve equilibrium, explaining the lower offset between diet and keratin. However, a recent study contradicted this slower growth rate during CR, reporting an increase in volume in certain hair types (e.g., guard hair) under CR.^96^ Although it is evident from the isotope data that hair growth occurred during CR in our study, the specific growth rate remains undetermined, especially considering that the inflection point (i.e., when body mass stabilized) was around 30 days for all groups.^93^ It is plausible that hair growth paused during the initial acclimation phase but resumed slowly after 30 days of CR for the most restricted groups leading to the lower diet-tissue offsets. Furthermore, it has been proposed that individual hairs grow longer and are shed less frequently in CR mice.^96^ Thorough investigation of hair growth during CR is imperative to interpret these data and stable isotope data from future studies.

The decrease in *δ*^15^N_ker_ values under CR could also stem from alterations in protein pools. Hair in the most restricted group might have used the dietary protein pool rather than relying on endogenous sources to synthesize keratin. This provides a potential explanation for the decrease observed between the control and the most restricted CR groups. This hypothesis is not mutually exclusive to the first, and these disparities could arise from a combination of tissue function and tissue formation effects.

A slight decrease outside of the analytical error is observed between the control and the most CR groups for *δ*^13^C_ker_ values (0.4 ‰) which is consistent with previous literature.^53^^,^^54^ During a fasting state, the body will begin to use its reserves and lower its expenditure to meet energy requirements. With CR, a greater mobilization of fat stores begins through gluconeogenesis (glucose synthesis) and β-oxidation (fatty acid oxidation).^66^^,^^97^ All of these different metabolic reactions lead to isotopic fractionation, inducing depletion in the *δ*^13^C values of co-forming tissues relative to diet.^98^ Our results here support previous analyses on the same individuals (e.g., a greater use of fatty acid *via* lipogenesis and β-oxidation compared to glycolysis as the level of restriction increased^99^). A shift from the carbohydrate metabolism to fatty acid oxidation was also observed in the same mice,^100^ which is also consistent with the decrease in *δ*^13^C_ker_ values. In addition to the fractionation during various metabolic reactions, the decrease in *δ*^13^C_ker_ values with extent of CR can be explained by the use of more ^13^C-depleted lipids *via* lipolysis as an energy source.^53^

The minor decrease of *δ*^34^S_ker_ values can be attributed to a decrease in methionine catabolism. During the feeding state, an increase in the rate of remethylation (methionine recycling) can be noted, whereas during the fasting state, the rate of transulphuration increases, i.e., methionine is mainly used for protein synthesis.^101^ Thus, mice that are not subject to CR (i.e., control group) would tend to recycle their methionine, which could induce isotopic fractionation and thus ^34^S-enriched tissues. On the other hand, mice subjected to CR would not recycle their methionine, a direct routing of this amino acid would take place for protein synthesis, which would result in tissues depleted in ^34^S compared to the control group.

### Variation in measured elemental content of keratin with extent of CR

With regards to the keratin data, the C, N, and S content (%) and C:N, N:S, and C:S data of all samples included in this study are typical of values reported for modern and archaeological human and animal hair^88^^,^^102^ and the theoretical composition of keratin.^103^ The sulfur content was slightly lower than the reported average but remained within the range of others.^104^^,^^105^ Although sample size is small (*n* = 8), and further experimental studies would be required, the results of the 0% group could perhaps be considered an empirical reference dataset for future studies on hair keratin. Based on these previous data, as well as those presented in this study, a guideline of %C 35–55 %, %N 10–20 %, and a wider range for %S of 1–7 % can be proposed.

The decrease in %S observed between the 40% and 30% groups was consistent with a previous study on sheep subjected to nutritional restriction where the %S in their wool decreased with restriction, as did cysteine and cystine content.^106^ The decrease in %S observed in the wool study was associated with a decrease in the proportion of high-sulfur proteins. This high-sulfur protein deficiency has also been noted in human hair in the case of malnutrition.^107^ As wool, hair, and fur all have the same composition and are comparable, it is therefore likely that this decrease in %S in the 40% group (and some members of the 30% group) is due to the same occurrence. However, given the larger standard deviation in the 30% group, caution should be taken regarding the effect of nutritional stress on %S in keratin and more research is needed.

### Variation in isotope ratios of bone “collagen” with extent of CR

The decrease in *δ*^13^C_coll_ values observed with extent of CR is consistent with the results of other studies,^48^^,^^53^^,^^108^^,^^109^ as well as for the hair keratin of this study. Regarding nitrogen, the reported magnitude of the increase of the *δ*^15^N values of proteins with CR has varied between studies depending mainly on the duration and severity of the restriction.^70^ The increase of 0.4 ‰ between the means of non-restricted and the most restricted mice in our study can be considered small compared to other studies,^110^ albeit within the range of findings from a meta-analysis that reported an increase of +0.5 ± 0.2 ‰.^70^ Furthermore, the absence of variations between the groups in terms of the medians required some caution. However, the measured differences observed here, between the 10% group and the most restricted groups (30% and 40%), would be explained by the recycling of ^15^N-enriched endogenous proteins to form new tissues as it has already been described in numerous studies.^53^^,^^69^^,^^110^^,^^111^ The hypothesis is validated by the increase of gluconeogenesis observed in the 40% group^99^ confirming that certain amino acids have been converted *via* gluconeogenesis into by-products to be used for energy. This small increase can also be imputed to the slow turnover rate of the cranium. Although, bone extract and diet might be close to equilibrium, a longer period of CR might lead to higher differences *δ*^15^N values between CR and non-CR mice.

While variation is detected for carbon and nitrogen in the bone extract, the absence of isotopic variation for sulfur may be attributed to the exclusive influence of the methionine metabolic pathway within bone collagen for this element (compared to many amino acids for carbon and nitrogen metabolic pathways), and therefore fractionation is likely to be rarer.^112^ Indeed, methionine is an essential amino acid, so it can only come from the diet. This means that there is a direct routing from the diet to bone collagen without participating in metabolic reactions that would induce isotopic fractionation.^113^

### Variation in measured elemental content of bone “collagen” with extent of CR

In contrast to the elemental compositions of keratin, the carbon, nitrogen, and sulfur content of bone “collagen” almost halved between the least and most calorie-restricted groups. All samples were prepared in randomized batches (with a mixture of individuals from different CR groups) and the reproducibility of the elemental analyser cannot explain this variation (i.e., the decrease being outside of the analytical error). Furthermore, proteomic analysis has confirmed that the extract was predominantly collagen, as expected following a Longin methodology.^56^ Although only around 40 % of the total samples were included for proteomic analysis, given the consistency of the results (and the fact that the Longin method is a well-established methodology), it is highly likely that “collagen” of similar protein content—but dominated by type-I collagen—was also extracted in the remaining samples as anticipated from the Longin method. The absence of correlation between the elemental content and the isotope values (except for the 10% CR group for carbon, which may be an artifact due to the small number of individuals) suggests that this decrease in elemental content might be due to the structure of the collagen itself.

The relative proportion of type-I collagen in the extract (compared to other proteins) was not influenced by CR. However, further research is needed at the peptide level, to see if the structure of the collagen is influenced in any way by CR, and whether these lower percentages of carbon, nitrogen, and sulfur contents are due to the increasing addition of other substances into the extracted fraction as measured in the elemental analyser. It has been shown that after a 60 % reduction of food, restricted rats showed a decrease in the formation of collagen cross-links.^114^ The decrease in C, N, and S elemental content in our study could be consistent with the related increase in other elements, such as oxygen (O) and hydrogen (H), due to an increase in hydroxyl bonds which are hydrophilic. Collagen fibrils can thus also provide an aqueous environment in which minerals can grow,^115^ and a less robust collagen structure could lead to a change in the behavior of collagen during the extraction process, creating an unanticipated bias in this routine preparation method with extent of CR. Indeed, a low %C and %N (albeit with C:N still within the acceptable range) has been observed with the production of salts during demineralization, especially if the acid is not completely washed out during that process.^72^ Calcium chloride (created from the reaction of hydrochloric acid and calcium phosphate in the mineral phase of the bone) can become trapped in collagen fibrils, adding mass to the extract, and therefore impacting the %C, %N, and %S values as determined during measurement by elemental analyser. A similar effect was observed in collagen samples from rat tendons which showed sodium acetate trapped in the collagen fibrils due to the neutralization of acetic acid by sodium hydroxide (NaOH).^116^ If salt precipitation is indeed the underlying cause, it can be postulated that the extent of salt precipitation during the modified Longin extraction protocol here is directly related to the quality of the *in vivo* tissue, which in itself is related to the extent of CR. In this scenario, the structural differences due to CR have likely introduced an unintended (and apparently graded) bias in the Longin extraction protocol with regards to the precipitation of mineral salts which then go on to influence elemental % composition as determined *via* elemental analysis.

### Impact for CR research and archaeological studies using stable isotopes

The results of this study align with the results of previous studies on the influence of CR on tissue carbon and nitrogen isotope ratios, and extend them, to include the influence on sulfur isotope ratios, and on % content of all three elements. Further, we included two sources of protein from the same individuals: hair keratin and bone “collagen”. The results of our study demonstrate that after only three months of CR, the first changes in carbon isotope values are noticeable in bone “collagen” as well as in hair keratin for nitrogen isotope values and varied to some extent in a graded manner. Although hair does not undergo remodeling and is supposed to reach equilibrium with diet faster than bone, changes in *δ*^13^C and *δ*^15^N values with the extent of CR were less obvious in this study than with the bone “collagen”. The influence of hair growth during CR needs further research and is central to better understand the decrease in *δ*^15^N_ker_ values measured.

In this study, although CR has affected the isotope values of carbon in a graded way, and to some extent isotope values of nitrogen in bone “collagen”, the magnitude of the *δ*^15^N increase is low (less than a trophic level). These findings suggest that these differences would not be enough to properly diagnose short-term dietary stress (i.e., less than three months) in the past. A study with a longer period of stress would be interesting to undertake to counteract the issue of bone collagen’s long turnover time and to ensure the complete equilibrium between tissues and diet. Although 40% CR might seem extreme, it is important to note that CR did not equal malnutrition here as mice under CR experiments are always monitored ensuring their good health. While, with malnutrition, a larger variation in the *δ*^15^N and *δ*^13^C values of bone “collagen” could be possible (as certain individuals might have experienced in the past during famines, for example), this is not confirmed by the results of the current study.

The significant decrease in %C, %N, and %S in the “collagen” of mice in relation to the level of restriction observed here is, however, confounding but also promising. Further research is required to understand more precisely the mechanism by which the extent of CR influences the elemental composition of bone collagen, which in this case has resulted in it deviating from the established range of values commonly used to assess the integrity of archaeological collagen. Here, we have postulated that CR may be affecting the structure of collagen formed during this physiological stress, resulting in poorly cross-linked collagen. In archaeological populations, collagen formed under similar circumstances could then be rejected based on the elemental content or C:N data while actually being of good quality (i.e., bearing isotopic data consistent with *in vivo* composition). The use of C:N is effective for filtering through contaminated or otherwise compromised archaeological samples, but it is conservative and, in this circumstance, and others, could lead to the rejection of bone extracts that are well-preserved collagen. While such false negatives, as well as false positives (where C:N is within the acceptable range but samples are known to be contaminated/altered), are a well-known phenomenon, various papers^57^^,^^117^ have called for the use of more expansive quality indicators (e.g., C:N, %CNS, amino acid composition, FTIR) to ensure the good quality of a sample. Here, we suggest that when the elemental content of a sample is outside of the quality range, coupled with an increase in *δ*^15^N values and decrease in *δ*^13^C values—or where there are other indicators of nutritional stress (e.g., linear enamel hypoplasia, stunted growth) —proteomic analysis should be integrated to further characterize the nature of the bone extract before isotopic data are rejected. The results of this study also highlight more broadly that elemental content of “collagen” can vary with diet (with nutritional stress in this case, or perhaps even with dietary content), and that further experiments should be carried out on the impact of diet on the elemental content of bone “collagen”. This will serve to verify if %C, %N, and %S in bone “collagen” might be used as a tool to identify potential cases of past nutritional stress, or indeed to re-evaluate the suitability of this commonly applied quality control criteria.

### Limitations of the study

As a retrospective isotopic study on material not initially intended for this purpose, there are a number of identified limitations. Hair was only analyzed at the end of the experiment, but ideally, it would have been collected throughout the study to see if isotopic changes would have been apparent in this tissue at an earlier phase of CR. Furthermore, analysis of the diet as a whole, and not by separating the different macronutrients, somewhat limits the interpretation of the different offsets between diet and tissue we explore here, particularly with regards to carbon. Ideally, future experiments should include parent-mice raised on an isotopically characterized (and constant) diet and then the subsequent experimental CR and isotope analysis of their litters (raised on that same diet both prior to and during the CR experiment) to limit diet-tissue equilibrium/turnover issues. Finally, our explanation about the change in collagen structure requires further analysis but is limited by remaining available material. Ideally, a study of the collagen structure at the peptide level would be undertaken, along with investigation of the bound water or salt content of the extracts.

## STAR★Methods

### Key resources table

**Table:** 

REAGENT or RESOURCE	SOURCE	IDENTIFIER
**Experimental models: Organisms/strains**		

C57BL/6	Charles River	N/A

**Deposited data**		

Proteomics and stable isotope data	This paper	Mendeley: https://doi.org/10.17632/z6246tggtv.1
Original code used for data processing and visualization	This paper	GitHub: https://github.com/Elea-Gutierrez/Short-termCR-isotopes.git

**Software and algorithms**		

R	https://www.r-project.org	3.6.3

**Other**		

Rodent diet used during the calorie restriction experiment	Research Diets, NJ, USA	D12450B: https://www.researchdiets.com/formulas/d12450b

### Resource availability

#### Lead contact

Further information and requests for resources and material should be addressed and will be fulfilled by the lead contact, Kate Britton k.britton@abdn.ac.uk.

#### Materials availability

Where sufficient material remains, the extracted bone “collagen” is available at the Archaeological Chemistry laboratory of the University of Aberdeen.

#### Data and code availability

• The generated data of the stable isotopes and proteomics analyses reported in this paper is available in this paper’s Supplemental Information 1 and publicly accessible at https://doi.org/10.17632/z6246tggtv.1 as of the date of the publication.
• The code generated during this study is available at https://github.com/Elea-Gutierrez/Short-termCR-isotopes.git.
• Any additional information required to reanalyse the data reported in this paper is available from the lead contact upon request.

### Method details

#### The CR study

Material analyzed here originates from a short-term (three months) CR study designed to characterize the response to CR in male C57BL/6 mice. A graded, 0 to 40%, CR protocol was used based on the positive linear response in lifespan with increasing levels of CR.^118^ The protocol has been described in detail elsewhere.^93^ All procedures were reviewed and approved by the University of Aberdeen Animal Welfare and Ethical Review Body, in accordance with the ARRIVE Guidelines.^119^

The mice were purchased between 10 and 12 weeks of age and acclimated for ∼1 week. Mice were individually housed at 20 ± 2°C. As mice are nocturnal, they were fed daily at lights out (18h30) with free access to water to avoid changes in their normal behavioral patterns. In the baseline phase (2 weeks prior to the introduction of CR), daily *ad libitum* food intake was measured to calculate the individual restricted rations. Mice were assigned to weight matched treatment groups.

Between seven and nine mice were exposed to one of five levels of CR: 0% (i.e., 12AL, *ad libitum* feeding for 12 h of darkness per day), 10%, 20%, 30%, and 40% lower calories than their individual *ad libitum* baseline intakes. All groups were fed the same diet throughout this study (D12450B: Research diets, NJ, USA) containing 20% protein, 70% carbohydrate, and 10% fat (by energy) (see key resources table). Samples of this homogeneous diet were retained for bulk isotopic analysis for the purposes of this study.

Following baseline, mice were calorie restricted at ∼140 days old (D140, age 5 months), when they reach full skeletal maturity.^120^ After three months of CR (D224; age 8 months), mice were killed by a terminal CO_2_ overdose. The components of the skeleton used in this study (cranium) were snap frozen in liquid nitrogen and stored at −80°C, while the pelage was removed, flattened, and stored at −80 °C at the University of Aberdeen, School of Biological Sciences. A total of 39 mice were available for analysis in this research.

No significant differences in body mass were found between the five restriction groups prior to initiating CR (D140; age 5 months). After three months of CR (D224; age 8 months), the final body mass had followed a graded response and was significantly lower in all CR groups compared to the 0% control. The inflection point, when body mass stabilized and mice reached energy balance, had a mean of 29.3 ± 2.09 days after CR started for all restricted groups.^93^

#### Preparation of samples for isotope analyses

Due to other study requirements (i.e., mechanical analysis), it was necessary to use the skull for bone collagen extraction and isotope analysis as it was the only skeletal element available in sufficient quantity and from the largest number of individuals. Parietal, interparietal, and frontal bones were already detached from the skull for brain harvest and were partially cleaned by the primary investigator during the CR experiment. Therefore, most tissues had already been removed. Prior to collagen extraction, the cleaning process of the removal of all remaining soft tissue from the skulls was achieved by low-temperature maceration in the School of Biological Sciences, University of Aberdeen. Each sample was placed in an incubator (Gallenkamp) at 30°C with deionized water to accelerate the maceration process without any risk of denaturation of the collagen. Bones remained in the incubator between eight and ten days until all tissues were removed. Once soft tissues had been successfully removed, remaining bones were left to dry under continuous airflow for 48 h.

Sample degreasing and collagen extraction were then undertaken in the Archaeological Chemistry laboratory at the University of Aberdeen. First, samples were degreased by following the methodology described in Guiry and colleagues.^121^ Bones were soaked in 2:1 chloroform/methanol solution in an ultrasonic bath twice for 15 min. The degreased samples were then rinsed in MilliQ water (18 MΩ.cm^−1^) for 10 min in an ultrasonic bath three times to ensure that the 2:1 chloroform/methanol solution has been removed. Finally, samples were left to dry under continuous airflow for 48 h. Collagen was then extracted from dry bones based on a modified Longin method^56^ following the steps outlined in Britton and colleagues^122^ without the ultrafiltration step. Between 31 mg and 65 mg of bone from each individual were soaked in 0.5 M of refrigerated hydrochloric acid (HCl) for demineralization. After 20 min, samples were demineralized (i.e., transparent, soft, and floating at the surface), removed from the acid, and rinsed with MilliQ water (18 MΩ.cm^−1^) before being gelatinized in a weak HCl solution (pH 3) in a heater block. Although other proteins than collagen are extracted during the Longin methodology,^56^ collagen is the main protein extracted and this extracted protein is referred to as “collagen” in this paper. Samples were then E-zee filtered, lyophilized, and weighed out into tin capsules.

Hair was harvested directly from the mouse skin by cutting the strands (∼0.5–0.7 cm) as closely as possible to the skin with dissection scissors. Sample size was between 26 mg and 79 mg for each individual. The same degreasing protocol used for the bones was undertaken, including rinsing steps, before hair was frozen, lyophilized, and sent for analysis.

Stable carbon, nitrogen, and sulfur isotope measurements for bone and hair were undertaken on a Delta V Advantage continuous-flow isotope ratio mass spectrometer coupled *via* a ConfloIV to an IsoLink elemental analyser (Thermo Scientific, Bremen) at the SUERC Radiocarbon Laboratory, East Kilbride, Scotland. The International Atomic Energy Agency (IAEA) reference materials USGS40 (L-glutamic acid, *δ*^13^C_VPDB_ = −26.39 ± 0.04 ‰, *δ*^15^N_AIR_ = −4.52 ± 0.06 ‰) and USGS41a (L-glutamic acid, *δ*^13^C_VPDB_ = 36.55 ± 0.08 ‰, *δ*^15^N_AIR_ = 47.55 ± 0.15 ‰) were used to normalize *δ*^13^C and *δ*^15^N values. Two in-house standards (GS2, *δ*^34^S_VCTD_ = −9.38 ± 0.24 ‰ and GAS2, *δ*^34^S_VCTD_ = 19.00 ± 0.20 ‰) that are calibrated to the International Atomic Energy Agency (IAEA) reference materials IAEA-S-2 (silver sulphide, *δ*^34^S_VCTD_ = 22.62 ± 0.08 ‰) and IAEA-S-3 (silver sulphide, *δ*^34^S_VCTD_ = −32.49 ± 0.08 ‰) were used to normalize *δ*^34^S values. Results are reported as permil (‰) relative to the internationally accepted standards VPDB for carbon, AIR for nitrogen, and VCDT for sulfur.^123^

Normalization was checked using an in-house collagen standard (DHB2019, *n* = 8, long-term average: −21.23 ± 0.12 ‰, 3.70 ± 0.11 ‰, and 9.50 ± 0.41 ‰), which gave values of −21.22 ± 0.04 ‰ (1 s d), 3.61 ± 0.08 ‰ (1 s d), and 9.66 ± 0.33 ‰ (1 s.d.) for *δ*^13^C, *δ*^15^N, and *δ*^34^S respectively, and the IAEA reference materials USGS88 (marine collagen, *n* = 4, −16.06 ± 0.07 ‰, 14.96 ± 0.14 ‰, and 17.10 ± 0.44 ‰), and USGS89 (porcine collagen, *n* = 4, −18.13 ± 0.11 ‰, 6.25 ± 0.12 ‰, and 3.86 ± 0.56 ‰), which gave values of −16.13 ± 0.07 ‰ and −18.10 ± 0.11 ‰ (1 s.d.), 15.21 ± 0.17 ‰ and 6.20 ± 0.11 ‰ (1 s d), and 17.27 ± 0.51 ‰, and 4.59 ± 0.28 ‰ (1 s d) for *δ*^13^C, *δ*^15^N, and *δ*^34^S respectively.^123^ Results are reported as permil (‰) relative to the internationally accepted standards VPDB (carbon), AIR (nitrogen), and VCDT (sulfur). The analysis of the samples in duplicate allowed us to calculate the mean analytical error, which was routinely 0.3 ‰ or better across all analyses. This is in agreement with long-term machine standard error (i.e., analytical error) in this laboratory, which is ±0.2 ‰ for *δ*^15^N values, ±0.1 ‰ for *δ*^13^C values, and ±0.4 ‰ for *δ*^34^S values.^123^ As the elemental analyser is not configured to control long term reproducibility, the standard errors of the check standards for each element have been used to control the analytical error which was for bone collagen ±0.3 % for %N and %C values, ±0.04 % for %S values, and for hair keratin ±0.1 % for %N and %S values, and ±0.5 % for %C values.

#### Proteomic analysis

Following the generation of the elemental and stable isotope data, proteomic analysis was carried out on a subsample (15 out 39) to confirm that the bone extract following the Longin method was indeed collagen. The samples were diluted in 50 μL of 6.0 M Gu-HCl, 200 mM Tris-HCl pH 8.5, 1 mM TCEP, 1.5 mM Chloroacetamide by probe sonication and heating to 95°C for 5 min, initially digested with LysC (Wako) with an enzyme to substrate ratio of 1/200 for 2 h at 37°C. Subsequently, the samples were diluted 10-fold with water and digested with porcine trypsin (Promega) at 37°C overnight. Samples were acidified to 1 % TFA, cleared by centrifugation (16,000 xg, 5 min at RT) and desalted using homemade C18 stage-tips. Peptides eluted using 50 % acetonitrile were lyophilized, resuspended in 0.1 % TFA/water and the peptide concentration was measured by A280 on a nanodrop instrument (Thermo). The sample was diluted to 1 μg/5 μL for mass spectrometry analysis.

Five μL of the resuspended peptides were analyzed by reversed-phase nano-LC–MS/MS using a nano-Ultimate 3000 liquid chromatography system coupled to Fusion Lumos mass spectrometer (both Thermo Fisher Scientific). Flow-rate used was 400 nL/min. Peptides were loaded onto a C18 capillary column (1.6, 0.075 mm × 15 cm; IonOpticks, Australia) and separated using a 67-min gradient: Buffer A, 2 % acetonitrile 0.5 % Acetic Acid, Buffer B, 80 % acetonitrile, 0.5 % Acetic Acid; (0–16 min: 2 % buffer B, 16–56 min: 3–35 % buffer B, 56–62 min: 99 % buffer B; 62–67 min 2 % buffer B. The Fusion Lumos was operated in top-12, data-dependent mode with a 30-s dynamic exclusion range. Full-scan spectra recording in the Lumos was in the range of *m*/*z* 350 to *m*/*z* 1,650 (resolution: 70,000; AGC: 3e^6^ ions). MS2 was performed with an isolation window of 1.4, AGC 5e^4^, HCD collision energy of 26, Scan range from 140 to 200 ms maximum injection time. The Lumos was operated in data-dependent mode with a 10-s dynamic exclusion range. Full-scan spectra recording in the Lumos was in the range of *m*/*z* 350 to *m*/*z* 1400 (resolution: 240,000; AGC: 7.5e5 ions). MS2 was performed in the ion trap, isolation window 0.7, AGC 2e^4^, HCD collision energy of 28, rapid scan rate, Scan range 145–1,450 *m*/*z*, 50 ms maximum injection time and an overall cycle time of 1 s.

#### Quantification and statistical analysis

All the statistical treatments and analyses of data were undertaken using R (Version 3.6.3). Data are expressed as median unless otherwise stated. Isotopic data were not normally distributed (confirmed by Shapiro-Wilk and Bartlett tests). Unless otherwise stated, Kruskal-Wallis tests were used to compare data between groups. Where appropriate, following Kruskal-Wallis tests, post-hoc Dunn tests were used with a Bonferroni correction to reduce Type-I errors due to repeated pairwise comparisons. A α of 0.05 was determined and the null hypothesis was rejected if the *p*-value ≤ α. For the post-hoc Dunn test, it is recommended to reject the null hypothesis if the *p*-value ≤ α/2.^124^
