# Supplementary material for: Carbon, nitrogen, and sulfur elemental and isotopic variations in mouse hair and bone collagen during short-term graded calorie restriction
Source: iScience. 2024 May 22;27(6):110059. doi: 10.1016/j.isci.2024.110059 (PMC11214416; doi:10.1016/j.isci.2024.110059)
Supplement: Document S1. Figures S1–S4 and Tables S1–S3 [file mmc1.pdf]

iScience, Volume *27*

Supplemental information

Carbon, nitrogen, and sulfur elemental and isotopic variations in mouse hair and bone collagen during short-term graded calorie restriction

Eléa Gutierrez, Sharon Mitchell, Catherine Hambly, Kerry L. Sayle, Alex von Kriegsheim, John R. Speakman, and Kate Britton

Gutierrez et al. Carbon, nitrogen, and sulfur elemental and isotopic variations in mouse hair and bone collagen during short-term graded calorie restriction.

Supplementary Information 1: Data tables

Supplementary Table 1: Isotopic data for all the individuals distributed by calorie restricted groups and mass gain between start and end of the calorie restriction experiment for the control group. Individuals highlight in grey did not pass the accepted quality criteria for C:N_{coll} according to Ambrose[1]. Data for the mass gain were obtained from Mitchell *et al.*[2]. Related to Table 1.

Sample name	CR group	$\delta^{13}C_{coll}$ (‰)	$\delta^{15}N_{coll}$ (‰)	$\delta^{34}S_{coll}$ (‰)	$\delta^{13}C_{ker}$ (‰)	$\delta^{15}N_{ker}$ (‰)	$\delta^{34}S_{ker}$ (‰)	Mass difference (g)
ST-16	0%	–20.6	7.2	7.6	–21.9	9.9	9.7	1.00
ST -17	0%	–20.8	6.7	8.2	–23.2	7.7	7.4	2.64
ST-18	0%	–20.7	6.9	7.6	–23.2	7.7	7.7	1.27
ST-20	0%	–20.7	7.2	7.5	–23.1	8.1	7.8	0.63
ST-31	0%	–20.7	7.5	7.2	–21.9	9.2	9.1	1.46
ST-32	0%	–20.8	7.5	7.9	–23.1	8.1	8.2	3.91
ST-38	0%	–20.9	6.6	8.2	–22.6	8.7	8.7	1.38
ST-45	0%	–20.7	7.1	8.2	–23.0	8.1	7.9	4.90
ST-8	10%	–20.7	6.7	8.2	–22.8	8.5	8.4	
ST-9	10%	–21.0	6.6	8.4	–23.2	7.7	7.8	
ST-21	10%	–21.1	6.6	7.2	–23.1	7.9	7.9	
ST-33	10%	–21.1	6.8	7.6	–22.2	9.3	9.3	
ST-46	10%	–20.9	7.6	7.2	–22.7	7.9	8.7	
ST-50	10%	–20.7	6.7	8.0	–23.0	8.0	7.9	
ST-54	10%	–21.1	6.8	8.0	–22.7	8.6	8.7	
ST-56	10%	–20.7	6.8	8.3	–21.8	9.8	9.8	
ST-4	20%	–20.8	7.1	8.9	–23.4	7.3	7.4	
ST-27	20%	–21.3	6.1	7.8	–23.2	6.8	7.4	
ST-37	20%	–21.3	6.5	7.2	–23.4	6.6	7.0	
ST-39	20%	–20.9	8.6	7.9	–22.8	8.4	8.4	
ST-47	20%	–21.0	7.0	7.5	–23.2	7.7	8.0	
ST-57	20%	–21.1	7.1	7.4	–23.0	7.6	7.8	
ST-64	20%	–21.1	6.8	7.4	–22.9	7.8	8.0	
ST-6	30%	–21.3	7.3	6.4	–23.3	7.4	7.4	
ST-24	30%	–21.0	9.6	7.3	–22.9	7.7	7.9	
ST-36	30%	–21.1	6.9	7.3	–22.6	8.0	8.1	
ST-49	30%	–21.2	7.1	8.1	–23.0	7.6	7.8	
ST-52	30%	–21.5	8.1	7.9	–22.9	8.0	8.2	
ST-53	30%	–21.3	6.9	7.4	–22.8	8.0	7.9	
ST-55	30%	–21.4	8.3	7.9	–23.2	7.5	7.7	
ST-7	40%	–21.1	7.7	7.6	–23.0	7.7	7.9	
ST-28	40%	–21.5	7.5	8.4	–23.0	7.8	7.9	
ST-30	40%	–21.2	6.8	7.2	–23.4	7.4	7.5	
ST-34	40%	–21.4	8.2	7.6	–23.3	7.3	7.4	
ST-44	40%	–21.4	7.0	7.2	–23.1	7.9	7.9	
ST-48	40%	–21.6	8.9	6.9	–23.0	8.0	8.0	
ST-58	40%	–21.4	7.2	8.4	–23.1	7.5	7.8	
ST-62	40%	–21.3	6.7	6.9	–23.0	7.8	8.0	
ST-67	40%	–21.7	7.2	7.0	–23.2	7.8	8.1	

Supplementary Table 2: Hair elemental data for all the individuals distributed by calorie restricted groups. Related to Table 2.

Sample name	CR group	$\%N_{ker}$	$\%C_{ker}$	$\%S_{ker}$	$C:N_{ker}$	$N:S_{ker}$	$C:S_{ker}$
ST-16	0%	14.6	45.4	3.5	3.6	9.7	35.2
ST-17	0%	14.4	46.2	3.5	3.7	9.4	35.1
ST-18	0%	14.6	44.5	3.5	3.6	9.4	33.7
ST-20	0%	14.4	45.3	3.4	3.7	9.6	35.5
ST-31	0%	14.2	46.9	3.7	3.9	8.9	34.2
ST-32	0%	14.6	43.9	3.6	3.5	9.4	32.9
ST-38	0%	14.5	46.2	3.5	3.7	9.6	35.7
ST-45	0%	11.7	36.0	2.7	3.6	9.9	35.7
ST-8	10%	14.0	43.9	3.4	3.7	9.4	34.4
ST-9	10%	14.3	44.1	3.5	3.6	9.4	33.8
ST-21	10%	14.0	46.3	3.4	3.9	9.3	36.0
ST-33	10%	14.5	45.8	3.6	3.7	9.2	33.8
ST-46	10%	14.2	46.0	3.6	3.8	9.0	33.9
ST-50	10%	14.3	45.0	3.5	3.7	9.2	33.9
ST-54	10%	14.5	45.4	3.8	3.6	8.7	31.8
ST-56	10%	14.6	45.2	3.7	3.6	9.0	32.5
ST-4	20%	14.6	44.0	3.2	3.5	10.6	37.3
ST-27	20%	14.6	44.8	3.5	3.6	9.4	33.7
ST-37	20%	14.7	46.1	3.7	3.7	9.0	32.9
ST-39	20%	14.5	45.1	3.5	3.6	9.4	34.3
ST-47	20%	14.6	44.5	3.6	3.5	9.4	33.2
ST-57	20%	14.6	44.4	3.6	3.5	9.2	32.5
ST-64	20%	14.3	44.2	3.7	3.6	8.9	32.1
ST-6	30%	14.6	44.7	3.6	3.6	9.2	33.0
ST-24	30%	11.5	34.3	2.9	3.5	9.3	32.1
ST-36	30%	14.7	45.0	3.8	3.6	8.9	32.0
ST-49	30%	14.8	44.5	3.8	3.5	8.8	31.0
ST-52	30%	15.1	44.6	3.8	3.5	9.1	31.6
ST-53	30%	14.5	44.6	3.8	3.6	8.9	31.7
ST-55	30%	14.7	46.1	3.2	3.7	10.7	39.1
ST-7	40%	14.0	45.4	3.2	3.8	9.9	37.6
ST-28	40%	14.7	45.0	3.5	3.6	9.6	34.3
ST-30	40%	14.6	44.5	3.3	3.6	10.1	36.1
ST-34	40%	14.6	44.8	3.5	3.6	9.6	34.4
ST-44	40%	13.9	43.0	3.0	3.6	10.7	38.9
ST-48	40%	14.6	45.3	3.3	3.6	10.1	36.7
ST-58	40%	14.4	46.6	3.1	3.8	10.6	40.1
ST-62	40%	14.4	45.6	3.4	3.7	9.6	35.4
ST-67	40%	14.4	42.8	3.1	3.5	10.7	37.4

Supplementary Table 3: Bone elemental data for all the individuals distributed by calorie restricted groups and proportion of type-1 collagen (in %). Individuals highlight in grey did not pass the accepted quality criteria for $C:N_{coll}$ according to Ambrose[1]. Related to Table 2.

Sample name	CR group	$\%N_{coll}$	$\%C_{coll}$	$\%S_{coll}$	$C:N_{coll}$	$N:S_{coll}$	$C:S_{coll}$	Proportion of collagen type 1 (in %)
ST-16	0%	14.7	41.3	0.3	3.3	326	99	64
ST-17	0%	14.7	41.7	0.3	3.3	367	111	
ST-18	0%	13.4	37.9	0.3	3.3	383	117	92
ST-20	0%	13.5	38.0	0.3	3.3	335	102	81
ST-31	0%	14.2	38.4	0.3	3.1	338	107	
ST-32	0%	14.6	39.2	0.3	3.1	364	116	
ST-38	0%	14.8	42.1	0.3	3.3	324	97	
ST-45	0%	14.8	40.9	0.3	3.2	352	109	
ST-8	10%	14.9	42.3	0.3	3.3	384	116	
ST-9	10%	13.6	39.3	0.3	3.4	355	105	
ST-21	10%	10.6	29.6	0.2	3.2	326	100	
ST-33	10%	11.8	32.7	0.2	3.2	356	110	68
ST-46	10%	13.7	35.0	0.3	3.0	293	98	
ST-50	10%	13.9	39.6	0.3	3.3	379	115	0
ST-54	10%	11.8	33.6	0.3	3.3	335	100	
ST-56	10%	13.1	37.1	0.3	3.3	369	111	
ST-4	20%	11.9	33.9	0.3	3.3	331	99	
ST-27	20%	14.1	39.9	0.3	3.3	333	101	80
ST-37	20%	12.7	35.1	0.3	3.2	369	114	
ST-39	20%	10.0	23.0	0.1	2.7	444	166	62
ST-47	20%	11.3	29.9	0.2	3.1	415	134	
ST-57	20%	11.3	30.4	0.2	3.1	399	127	
ST-64	20%	9.9	26.1	0.1	3.1	540	175	82
ST-6	30%	12.4	32.2	0.2	3.0	405	133	
ST-24	30%	10.7	24.2	0.2	2.6	401	153	75
ST-36	30%	11.7	31.7	0.2	3.2	484	153	
ST-49	30%	9.4	25.4	0.2	3.1	396	126	
ST-52	30%	8.9	20.8	0.1	2.7	458	167	61
ST-53	30%	10.2	25.6	0.1	2.9	468	159	84
ST-55	30%	10.8	26.5	0.2	2.9	445	155	
ST-7	40%	8.4	19.8	0.1	2.8	461	167	75
ST-28	40%	9.6	22.8	0.1	2.8	524	189	
ST-30	40%	12.4	33.5	0.2	3.2	461	146	
ST-34	40%	10.6	24.7	0.2	2.7	422	155	89
ST-44	40%	9.5	25.3	0.1	3.1	532	172	
ST-48	40%	10.7	23.8	0.1	2.6	475	183	78
ST-58	40%	7.3	18.4	0.1	2.9	522	178	
ST-62	40%	8.8	24.0	0.1	3.2	499	156	
ST-67	40%	11.2	27.5	0.2	2.9	488	171	

Supplementary Information 2: Figures

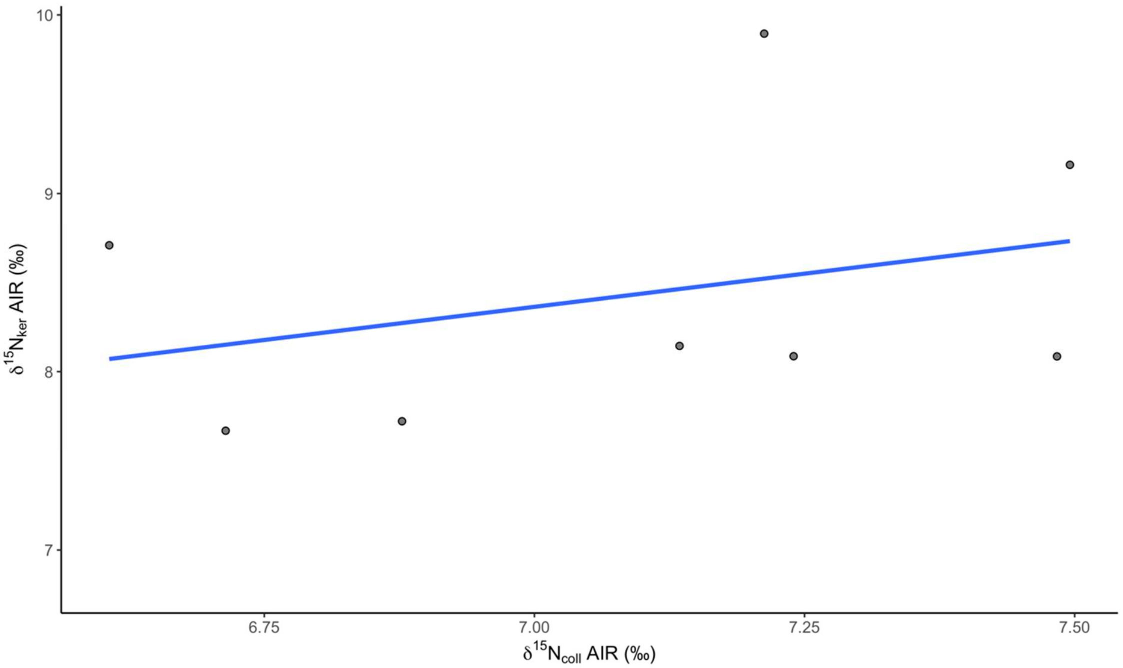

Figure S1. Comparison of nitrogen isotopic values of pair hair keratin and bone extract from mice fed *ad libitum*. Regression line shown in blue. Related to Table 1.

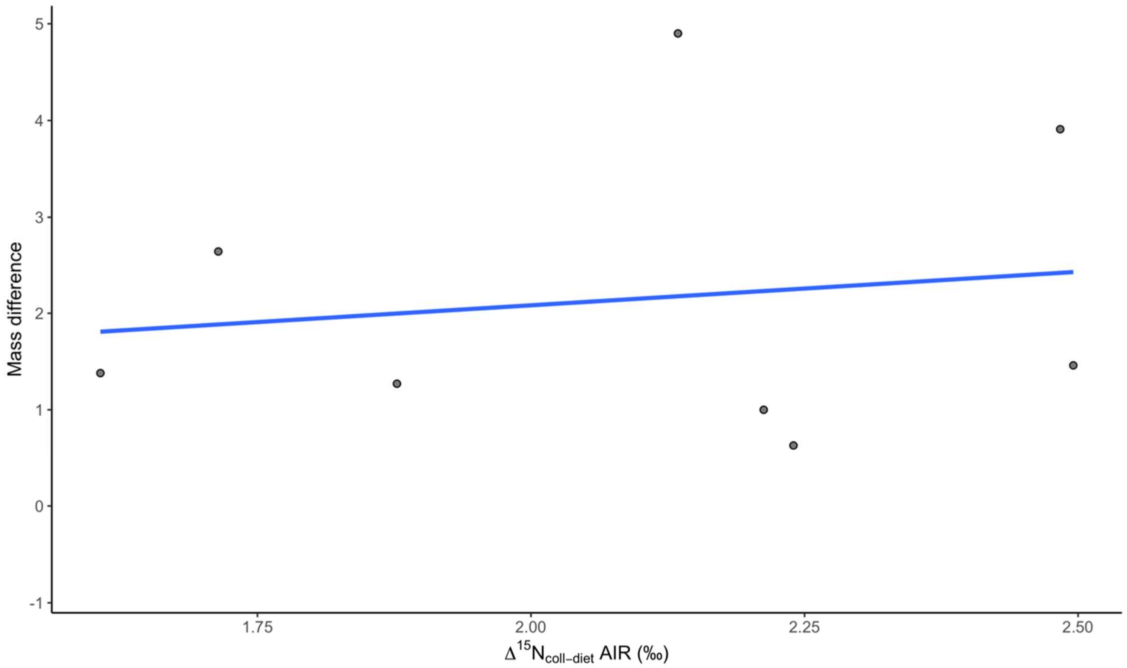

Figure S2. Comparison of nitrogen isotopic offset between diet and bone protein and mass gain between start and end of the calorie restricted experiment from mice fed *ad libitum*. Regression line shown in blue. Data for the mass gain were obtained from Mitchell *et al* [2]. Related to Table 1.

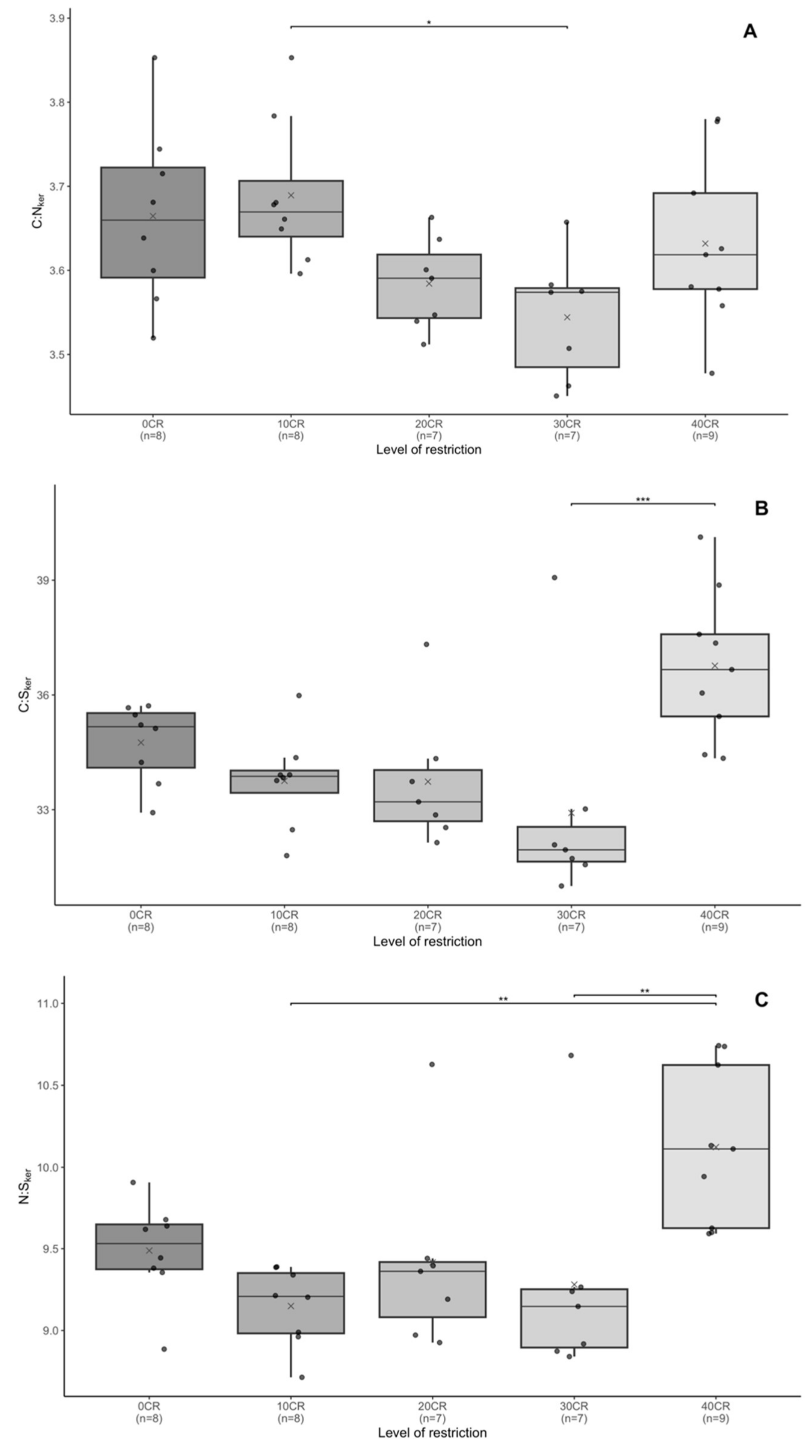

Figure S3. Distribution of the molar ratio of hair keratin from mice undergoing three months graded calorie restriction (CR). Mice were restricted by 0-40% *ad libitum* intake. The grey crosses indicate the mean values for each group, and the horizontal lines through the boxes represent the median. *, **, and *** indicate a significant difference between groups with a *p*-value <0.025, <0.01, and <0.001 respectively. A.) Carbon-to-nitrogen ratio (C:N) of hair keratin. B.) Nitrogen-to-sulfur ratio (N:S) of hair keratin. C.) Carbon-to-sulfur ratio (C:S). Related to Table 2.

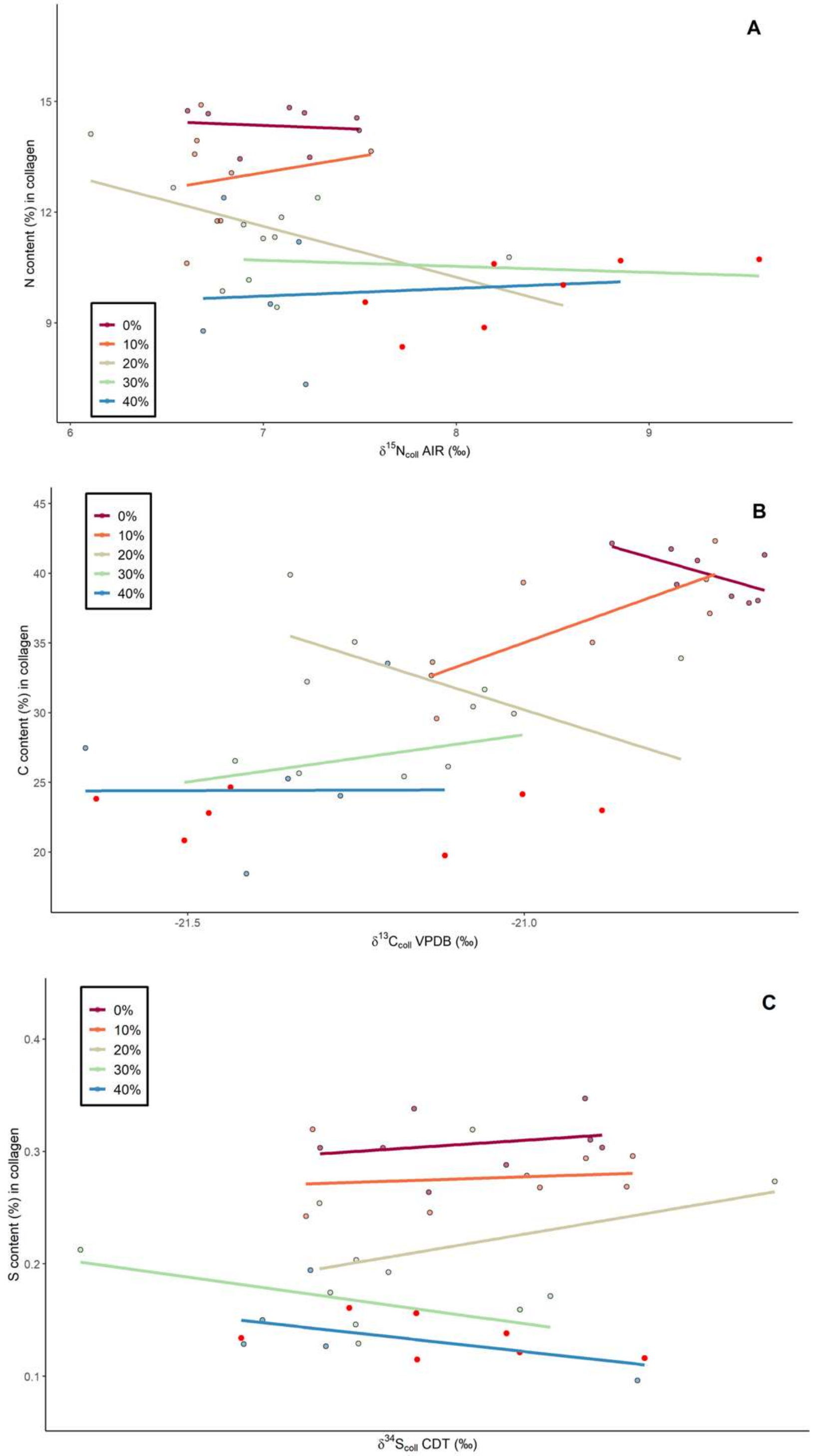

Figure S4. Comparison of isotopic values and elemental content from mice undergoing three months graded calorie restriction (CR). Mice were restricted by 0-40% *ad libitum* intake. The red dots represent mice (included in the analysis) that did not meet the accepted quality criteria for $C{:}N_{coll}$ according to Ambrose[1]. A.) Comparison of isotopic values and elemental content for nitrogen. B.) Comparison of isotopic values and elemental content for carbon. C.) Comparison of isotopic values and elemental content for sulfur in C. Related to Tables 1 and 2.

Supplementary Information Reference List

1. Ambrose SH: (1990). Preparation and characterization of bone and tooth collagen for isotopic analysis. J Archaeol Sci 17(4), 431-451. https://doi.org/10.1016/0305-4403(90)90007-R

2. Mitchell SE, Tang Z, Kerbois C, Delville C, Konstantopedos P, Bruel A, Derous D, Green C, Aspden RM, Goodyear SR, Chen L, Han JJ, Wang Y, Promislow DE, Lusseau D, Douglas A, Speakman JR (2015). The effects of graded levels of calorie restriction: I. impact of short term calorie and protein restriction on body composition in the C57BL/6 mouse. Oncotarget 6(18), 15902-15930. 10.18632/oncotarget.4142
